# A cross sectional observational study on the effect of music on the anxiety state of patients admitted for COVID 19 in a tertiary care hospital in New Delhi

**DOI:** 10.1192/j.eurpsy.2022.1237

**Published:** 2022-09-01

**Authors:** N.A. Jose, R. Prakash, P. Bansal, M. Khan

**Affiliations:** Hamdard Institute of Medical Sciences & Research, Psychiatry, New Delhi, India

**Keywords:** Covid-19, Anxiety, music

## Abstract

**Introduction:**

COVID 19 has led to dramatic changes in the lives of people leading to an increase in stress and anxiety. Music intervention is a non-medicated method for relieving anxiety. This current study aims to understand whether music can be effectively used to alleviate anxiety in admitted COVID-19 patients.

**Objectives:**

To study the effect of music on anxiety in patients admitted for COVID-19 in a tertiary care hospital in New Delhi

**Methods:**

34 patients (17 females &17 males) were randomly divided into two groups, a control (N=17) and a music group (N=17). Vitals of all the patients were noted. Patients of the music group were asked to listen to relaxing instrumental for 30 minutes, while patients of the control group were asked to relax for 30 minutes. Vitals of all the patients were noted again. Patients were asked to fill State Trait Anxiety Inventory (STAI) before and after intervention.

**Results:**

The post-intervention mean scores of STAI after the music session were lower in the music group than the control group [95.06 (SD 8.5)) versus 102.37 (SD 10.3)]. The differences in mean values of pre- to post-intervention changes between both groups after music session were statistically significant.

**Conclusions:**

Our findings suggest that listening to music lowers anxiety. As music is non- invasive and free of side-effects we recommend that music intervention service should be used to improve health care quality.

**Disclosure:**

No significant relationships.

